# Narcotics Anonymous attendees’ perceptions and experiences of substitute behaviors in the Western Cape, South Africa

**DOI:** 10.1186/s13011-023-00552-z

**Published:** 2023-07-05

**Authors:** Deborah Louise Sinclair, Steve Sussman, Shazly Savahl, Maria Florence, Wouter Vanderplasschen

**Affiliations:** 1 Department of Psychology, University of the Western Cape, Cape Town, 7535 South Africa; 2 Department of Special Needs Education, Ghent University, 9000 Ghent, Belgium; 3 Departments of Population and Public Health Sciences, and Psychology, and School of Social Work, University of Southern California, Los Angeles, CA 90032-3628 USA; 4 Centre for Interdisciplinary Studies of Children, Families and Society, University of the Western Cape, South Africa, Cape Town, 7535 South Africa

**Keywords:** Substitute behaviors, Recovery support groups, Substance use, Behavioral addictions

## Abstract

**Background:**

Much remains unknown about the dynamics of substitute behaviors during addiction recovery among persons attending recovery support groups. Insight into the nature, motives for, and course of substitute behaviors could help to shape recovery support and harm reduction services.

**Methods:**

Twenty-three semi-structured in-depth interviews (*n* = 14 males and *n* = 9 females) were conducted with a convenience sample of Narcotics Anonymous attendees from a number of groups in the Western Cape, South Africa. Participants ranged in age from 22—55 years (M = 39.3, SD = 9.35).

**Results:**

Thematic analysis yielded four themes: (i) substance-to-substance substitution; (ii) substance-to-behavior substitution; (iii) substitute behaviors and harm (reduction) and (iv) support needs to manage and resolve substitute behaviors. According to the study, participants’ substitute behaviors developed across recovery stages; were temporary or long-term replacements for substance use disorders and were engaged for distraction, isolation from others, calming, assuaging boredom, keeping occupied, filling a perceived experiential void, modifying mood and to self-medicate. While substitutes were utilized for harm reduction or relapse prevention, the potential for ostensibly healthy behaviors to threaten recovery and lead to relapse was also recognized.

**Conclusions:**

Self-monitoring, ongoing vigilance, and awareness of when substitutes become genuine addictions are critical for timely, suitable interventions.

**Supplementary Information:**

The online version contains supplementary material available at 10.1186/s13011-023-00552-z.

## Background

With a presence in 143 countries and approximately 76,000 weekly meetings, Narcotics Anonymous (NA) is one of the largest global fellowships of persons in recovery from substance use disorders (SUDs) [1, 2]. NA, alongside other 12-Step programs (e.g., Alcoholics Anonymous, AA), represents a peer-assisted pathway to recovery [3] and is a well-established adjunct or alternative to formal treatment [4, 5]. Addiction recovery has been defined as “a voluntarily maintained lifestyle characterized by sobriety, personal health, and citizenship”, ([6], p. 222). 12-Step involvement has been shown to facilitate continuous abstinence and remission, as well as to confer recovery-supportive benefits including connectedness, support, acceptance, and enhanced quality of life [5–9]. However, meeting attendance may be hindered by a perceived poor fit, negative experiences within NA, becoming established within NA but not mainstream society [10], and refusal to accept its key tenets [11]. While the 12-Step fellowship itself is intended to be “vastly more than” a “sufficient substitute” for abstained addictive behaviors ([12], p. 152), and NA is premised on “powerlessness over a process of addiction rather than powerlessness over a particular substance” ([9], p. 2), substitute behaviors may nonetheless often be experienced by persons in recovery and precipitate relapse [13–16]. Substitute behaviors here refer to the newly acquired or resumed use of substances and engagement in behaviors that (partially or fully) functionally replace a terminated SUD [17].

Factors contributing to relapse include the availability and accessibility of substances; boredom; surplus money; lack of purposeful activities and structured time; loneliness; and substituting one addiction for another [17–21]. Although not all persons in recovery substitute a SUD [22], substitute addictions frequently occur [23]. While some have argued that substitutes are a potentially less harmful alternative to SUD (i.e. harm reduction), the negative impact on the recovery process remains as they may ultimately lead to relapse or the development of an equally or more harmful behavior even when used instrumentally [16, 23].

Though there has recently been a renewed research interest in substitute behaviors (e.g. [24]), the vast majority of studies employ quantitative methods and have been undertaken in the USA, starting at least as early as the 1950s. Little is known about other addictive behaviors as a replacement for substance use [17]. Moreover, an abundance of these studies, mirroring the recovery literature more broadly, has centered on those in early recovery (less than one year, [6]). To further elucidate “when and for whom this concept applies” ([22], p. 176), those in sustained (between one and five years) and stable recovery (more than five years [6];) can also offer critical insights into the occurrence of substitute behaviors, course of involvement in substitutes, and how the use of substances or engagement in behaviors relate to recovery and reduce or confer harm.

Addiction Interaction Disorder [25] theory suggests that the interplay between co-existing addictive behaviors may manifest in distinct patterns. Concurrent addictions interact, support, and join each other resulting in an addiction set that is more harmful than individual addictive behaviors. Of these configurations, two relate to substituting: replacement (one addiction is exchanged for another) and alternating addiction cycles (the dominant addiction shifts in a pattern) [25, 26].

In low- and middle-income countries (LMICs) such as South Africa, substitute behaviors are a growing concern given the limited availability of SUD treatment, which restricts opportunities to re-enter treatment if one relapses [27]. The substance use treatment system in the country mirrors the public–private dichotomy in health care [28]. Consequently, there is a strong reliance on state-funded treatment services, and with it long waiting times for treatment [29]. It is within this system that recovery support groups such as NA play a critical role [23], particularly as aftercare services are so limited [30, 31]. The approximately 350 face-to-face NA weekly meetings in all provinces are a testament to the widespread, international adoption of the program [32]. Yet, research on peer-assisted recovery in general, and NA in particular, is limited especially in LMICs [33]. There is an identified need to elucidate NA members’ recovery experiences [7]. Understanding NA members’ recovery experiences is important as lived experience affords “an increase in practical knowledge of addiction and recovery, empowerment, hope, and community connectedness” ([34], p. 232) and can be leveraged to benefit others seeking recovery. In these and other contexts, NA attendees represent an important and comparatively understudied population for improving our understanding of substitute and addictive behaviors. Greater knowledge of the topography, motives, and trajectory of substance and non-substance substitute behaviors throughout recovery stands to inform treatment and recovery support and harm reduction services in South Africa. Furthermore, as research on relapse rates and mechanisms is limited in the global South, and the attributes of people who relapse are poorly understood [35] there are a myriad of potential benefits to knowing more about substitute behaviors during recovery. Consequently, the purpose of this study is to explore NA attendees’ perceptions of and experiences with substitution in the Western Cape, South Africa. To the best of our knowledge, this is the first study undertaken with NA members on this topic.

## Materials and methods

As part of a broader multi-method study of the nature and dynamics of substitute addictions [23], this article employs an exploratory design within a qualitative methodological framework. We have applied the consolidated criteria for reporting qualitative research (COREQ) checklist to this study [36]. Data for this study were collected through individual in-depth interviews guided by a semi-structured interview schedule. Specific substitute substances that were probed included alcohol; nicotine/cigarettes; CAT (methcathinone, ephedrine); cocaine/crack; cannabis; cannabis/Mandrax; ecstasy; heroin; inhalants; methamphetamine; Nyaope (a combination of cannabis, antiretroviral drugs, heroin, cocaine, opioids, and bulking/cutting agents) [37] /Whoonga (low-grade heroin), over-the-counter drugs, and prescription medicines such as Ritalin (methylphenidate). Potential substitute behaviors that were queried were exercise; shopping; sex; eating; work; love/ relationships; religious activities; use of the internet and video games; social networking (e.g., Facebook), and gambling (the focal addictions mentioned in [2]).

### Participants and sampling

Participants were recruited using purposive sampling and subsequently snowball sampling techniques. Inclusion criteria were current NA meeting attendance and being in self-defined recovery. As recovery support groups enforce anonymity and have a closedness to ‘outsiders’ [38], initial access resulted from referrals by AA members that were already recruited into the larger study for interviews. AA was contacted through its regional office, and the first author was connected to the *Cooperation with the Professional Community Chairperson* who became the first interviewee. While AA and NA are autonomous, links between the fellowships expressed by NA members in this study included initially attending both fellowships; belonging to one fellowship and then switching to another, or being sponsored by someone in AA that has been in recovery for a longer period. In contrast to AA, NA targets those seeking recovery from all substances, i.e., including but not limited to alcohol. The referral from AA was essential for gaining entry as well as establishing trust. After each completed interview, and to ensure that anonymity was upheld, participants were asked to explain to their potential referrals the purpose of the research and how data would be collected. Only those expressing willingness to participate were asked to provide contact details to be shared with the researcher, and these were then contacted. Participants’ narratives were included whether or not they believed that they had substituted their primary substance, as they could still reflect on their experiences of recovery and dynamics related to substitution within the recovery support group.

### Data collection

The interview schedule was developed after consultation with the available literature on substitution and refined by the research team. Time constraints did not permit pilot testing. Individual in-depth interviews (*n* = 23) were conducted at participants’ homes and workplaces, restaurants, and coffee shops. Nineteen interviews were conducted by the lead author and four interviews were led by a master’s student with input from the lead author between 18 October—16 December 2018. In the South African context, the lead author (DS, a cisgender female in her early thirties) is categorized as ‘Coloured’^1^ and from a lower middle-class background, while the co-interviewer (EM, a cisgender female from Belgium in her early twenties) would be described as ‘White’ and upper-middle-class. Both interviewers had postgraduate training in addiction science and at the time were Ph.D. and master’s students, respectively. DS is registered as a psychological counselor, has substance use counseling experience, and has considerable data collection experience with people who use(d) substances. Alongside her academic training, EM completed an internship at a private substance use treatment facility in South Africa. Participants were briefed on the purpose of the study, encouraged to ask questions for clarity, and then provided their written consent that they wanted to participate. Data were collected until saturation was reached. Interview sessions, which were conducted in English and Afrikaans, were audio-recorded, lasting between 31 and 157 min. Field notes were also made during and after the interview. The interview schedule was not formally translated into Afrikaans, but, when participants preferred, the questions were translated and clarified in Afrikaans. Participants ranged in age from 22-55 years (M = 39.3, SD = 9.35), of which 14 were male and 9 were female. Three participants were in early recovery (< 1 year), 10 were in sustained recovery (1-5 years) and 10 self-identified as being in stable recovery (5 > years). One participant identified as ‘Black’, 13 as ‘Coloured’, and nine as ‘White’, of whom three were non-South African citizens (Belgian, Dutch, and British, respectively) currently residing in the country, some for the express purpose of accessing recovery support. Four participants were divorced, eight were married and 11 were unmarried (one engaged). A description of the participants is provided in Table 1.

**Table 1:** Description of participants (*n* = 23)

Participant	Gender	Age	Race	Addictive behaviors	Time in recovery	Received treatment (outpatient/ residential)	Substitutes since entering recovery
1	F	50	White	Alcohol, cannabis, cocaine	4 years	No	Sex/relationships
2	F	36	Colored	Alcohol, ecstasy, Mandrax, cannabis, Crystal Methamphetamine, over-the-counter medication	10 years	Yes	Shopping; food; work; cigarettes; coffee
3	F	43	Colored	Cannabis, Mandrax, Crystal Methamphetamine, alcohol	16 years	Yes	Cigarettes; food
4	M	38	White	Heroin, Crystal Methamphetamine, pornography, video games	1 year and 8 months	Yes	Do not believe they substituted
5	M	55	Colored	Alcohol, heroin	9 years and 6 months	Yes	Cigarettes; sex/relationships, pornography; binge-watching
6	M	44	Colored	Crack cocaine, Crystal Methamphetamine, alcohol	8 years	Yes	Cigarettes
7	M	26	White	Prescription medication (Zolpidem, Ritalin®), crack cocaine, Crystal Methamphetamine, alcohol	3 months	Yes	Cigarettes; exercise
8	M	51	White	Sex cannabis, alcohol, cocaine	8 years and 11 months	Yes	Food
9	F	37	Colored	Crystal methamphetamine, cannabis	4 months	Yes	Food; shopping; cigarettes
10	F	52	White	Alcohol, caffeine, nicotine, crack together with Mandrax, sleeping pills	9 years	Yes	Exercise; cigarettes; coffee; work
11	M	39	White	Alcohol, cannabis	5 years and 2 months	Yes	Do not believe they substituted
12	M	31	Black	Alcohol, cocaine	3 years and 6 months	Yes	Do not believe they substituted
13	F	33	Colored	Drugs, alcohol, sex, and love addiction, food	7 years	Yes	Food
14	M	30	Colored	Heroin	4 years	Yes	Exercise; pornography; food
15	M	44	White	Crystal Methamphetamine	6 months	Yes	Cigarettes; coffee; pornography; food
16	F	26	Colored	Crystal Methamphetamine	2 years	No	Food; gambling; cigarettes; binge watching
17	M	30	White	Heroin and crack cocaine	3 months	Yes	Exercise
18	F	51	Colored	Cannabis, crack, cocaine, alcohol, hallucinogenics, cigarettes, ‘reckless spending’	14 years	No	Food
19	M	22	White	‘Anything I can get my hands on’, cannabis, alcohol, cocaine, ecstasy	3 years and 6 months	Yes	Stealing
20	M	34	Colored	Crystal Methamphetamine, Mandrax/cannabis	4 years	No	Work; sex (escorts, pornography)
21	M	40	Colored	Crystal Methamphetamine, sex, masturbation, and food	3 years	Yes	Exercise; food; vaping; pornography
22	M	46	Colored	Crack, Mandrax, alcohol	13 years	Yes	Do not believe they substituted
23	F	46	Colored	Alcohol and drugs	5 years	Yes	Cigarettes/ vaping; binge watching

### Data analysis

Audio recordings of interviews were transcribed verbatim and data were analyzed using reflexive thematic analysis by the first author [39]. This analysis focuses upon (1) behaviors or activities that were used or engaged repeatedly / more / had been initiated since coming into (abstinence-based) recovery; (2) perceived motives or factors that played a role in (potential) substitution occurring or not; (3) perceptions of substitute-related harm and (4) related recommendations for treatment services. The analysis entailed becoming acquainted with the data through transcription and repeated reading of the text. To ensure the accuracy of the transcription recordings were listened to while reading the typed transcript. To start identifying patterns within interviews, initial codes were assigned. Thereafter, excerpts that corresponded to emerging patterns were highlighted. Next, themes and sub-themes were generated and discussed with co-authors (WV and S.S. (Steve Sussman)) to reach an agreement on adequacy. To enhance the credibility and trustworthiness of the study, I (the first author) purposefully engaged in reflexivity. Ongoing, critical conversations helped me to disentangle and examine how my academic training in psychology and addiction care, experiences of witnessing substance use within my community, substance use counseling experience, and mentorship from addiction experts shaped my views of addiction and recovery. Finally, the findings were situated within the extant research literature.

## Results

**Fig. 1:**
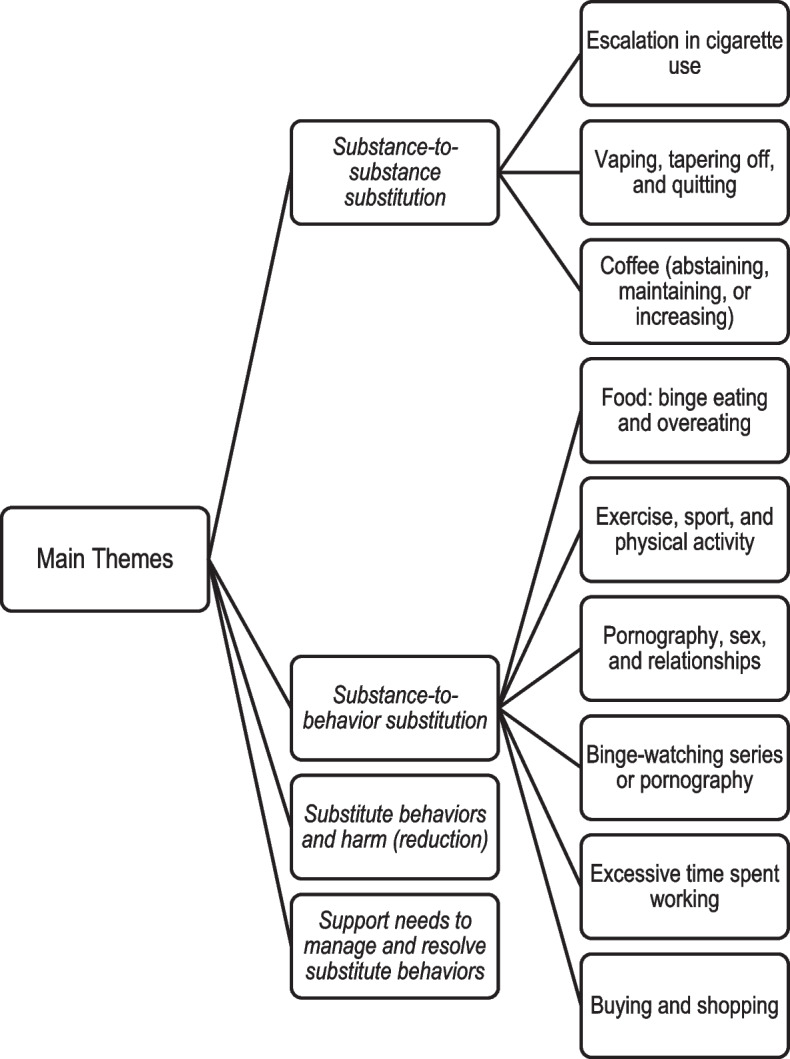
Themes and sub-themes. Four main themes and nine sub-themes emerged from the analysis

Four themes were identified within participants’ narratives: (1) substance-to-substance substitution; (2) substance-to-behavior substitution; (3) substitute behaviors and harm (reduction) and (4) support needs to manage and resolve substitute behaviors. The first two themes discuss the array of substitutes participants experienced and how these presented. The third theme explores its potential consequences for sustaining or hindering recovery, and the final theme foregrounds how substitutes may be prevented or managed. Nineteen participants believed that they had substituted for their SUD with substances or behaviors of varying severity since beginning their recovery journey. Illustrative quotes that best elucidate each theme are provided with accompanying participant numbers. The themes are visually depicted in Fig. 1.

## Substance-to-substance substitution

The leading substance-based replacements for a SUD among the selected NA participants were cigarettes and e-cigarettes (*n* = 11). Four patterns of use were identified: initiating smoking in recovery; maintaining cigarette consumption at the same level as in ‘active addiction’; escalating cigarette use and tapering off/ wanting to quit. Participants also reflected upon the acceptance of smoking during recovery. Another substance-based substitute identified by participants was coffee (*n* = 3).

People who did not use tobacco and nicotine products provided accounts of initiating smoking during recovery. The behavior may be maintained for a set time and then be abstained from, or, may endure.

Two years clean and I stopped using cigarettes […]. So, I didn't smoke even in active addiction, but in recovery, when I was in treatment they said like “I think you probably need to smoke” […]. I started smoking a bit more. Participant 5.

### Escalation in cigarette use

Several people who currently smoke observed an escalation in their cigarette consumption in recovery. Explanations included regulating anxiety and boredom and providing comfort.

I smoked more in the year after I stopped using. So, it was like a comfort and it was also when I was anxious and so it was just all the time […]. I used to smoke maybe five-six cigarettes a day in active (addiction). I was smoking 20 a day (in recovery). Participant 10.

### Vaping, tapering off and quitting

Some described efforts to quit due to adverse health consequences, or to reduce the number of cigarettes smoked. Efforts toward terminating use included reducing the number of cigarettes smoked per day as well as vaping. In the excerpts below, the combination of reduced cigarette consumption and vaping was regarded as less harmful than the initial addiction set. Not all quit attempts endured in the long term.

Cigarettes is one thing I’ve managed to cut down. I vape. […] And […] maybe six cigarettes a week […] I’d love to quit it completely, everything. And that’s what I’m working for, towards […] But I also don’t see smoking as a bad thing considering all the stuff I’ve dropped. Participant 12.

I quit for three years and then I started again and then I quit for 18 months and then I started again. So, I think my biggest problem is smoking cigarettes. Participant 3.

While respondents argued that illicit substances were more socially disapproved of and led to greater losses, the question was also raised of whether one was legitimately abstinent or in recovery, if cigarettes were mood-and-mind altering, as all drugs are.

Drinking too much coffee doesn’t make you push a trolley (become homeless) […], smoking cigarettes doesn’t ruin all your relationships […]. The hard drugs that do that. But get out there and swap it for all the other ones, uhm, to manage it. So then, are you actually really clean? […] A drug is a mood- or mind-altering substance […]. That cigarette alters my mood and my mind. Are we all in denial? […] 7 to 10-min smoke breaks at NA meetings? We go and smoke drugs. Participant 15.

### Coffee (abstaining, maintaining, or increasing)

A few participants increased their coffee consumption in recovery or maintained the quantity used in ‘active addiction’, except when forced to abstain.

I smoke cigarettes and drink a lot of coffee. They also think that's addiction but I'm not too hard on myself for that. Yes, one day I'm going to put it down; it's going to happen, […] but not now (laughs). Participant 2.

## Substance-to-behavior substitution

Shopping, exercise, food, binge-watching, gambling, work, as well as sex and relationships, emerged as common behavior-based substitutes for SUDs.

### Food: binge eating and overeating

Several participants (*n* = 10) reported binge eating and overeating. Food served many functions in recovery and overeating often included, but was not limited to highly palatable foods and those high in sugar and fat. For some, dysfunctional eating patterns were cyclical and long-standing. Binge eating may be used to manage fear, anxiety and discomfort and to avoid dealing with feelings.

I work on long binge-purge cycles, gain a lot of weight, eat very unhealthily, and then I lose it and I gain it again […]. The real problem behavior at the moment is food […]. I've got this picture of a blanky […]. I think a lot of ‘addicts’ have taken on a series of blankets. […] Our inability to connect with our true feelings […] something to pull over ourselves when we’re afraid […] nervous, […] uncomfortable, […] don’t want to deal with feelings. Participant 8.

As the drug of choice may be used to regulate eating and manage weight, abstinence may be associated with altered eating patterns and weight gain which can be distressing. Food may also be used to assuage boredom and to keep occupied.

Because I've now stopped my other addictions, it's progressed much faster and it's starting to bother me […]. A month ago, I ate so much that I […] had to vomit […] I don't wait until I get hungry. I just eat because it's the next thing—if I'm not smoking […] if I’ve got nothing to do, I'm eating. Which is terrible! […] It's one of the reasons […] I loved using tik (crystal methamphetamine), because it stopped me from compulsive eating […]. It made me thin […]. Gave me self-esteem, but also took everything else away from me […]. I kept using it, because I didn't want to end up like this […]. It's that horrible feeling inside that's killing me. Participant 9.

The preoccupation with and effects of the primary substance in ‘active addiction’ often led to food deprivation. In her recovery, however, this female participant consumed large quantities of chocolate and a high-sugar beverage (six litres daily) leading her to express concern about negative health impacts, some of which she was already experiencing.

I’m eating everything I see […]. You’re not smoking anymore so you’re eating […]. Gas cooldrinks. […] That is the biggest problem I have. Since I stopped smoking, oh my goodness. […] I had gastro now a few times; ulcer […]. Maybe three (2-L) Jive (soft drink brand) cooldrinks, alone […]. There must be a cooldrink in front of my bed. If I wake up in the night because I’m thirsty to drink, gas drink […]. This chocolate […] it’s almost like a drug to me now. Participant 16.

In another example, when querying a lengthy bank statement, a participant that considers their relationship with food to be problematic found that she had purchased fast food on at least 54 occasions in a given month. As she went on to describe, “drugs and alcohol merely need abstinence”, whereas food (and sex) require management. A task in recovery was thus to establish *how to* eat healthily.

I had a nine-page bank statement for the month […]. I found that I went to KFC for 54 swipes that month. Now, that excludes the amount I paid cash […]. More than twice a day, obviously sometimes three or four times a day […]. That’s ludicrous. […] I’ve got a very very toxic relationship with food. Participant 13.

### Exercise, sport and physical activity

Some participants (*n* = 5) engaged in exercise for the range of mental and physical benefits it conferred. One participant, who did not believe that he had substituted his addiction with exercise, recognized that he needed to remain vigilant to crossing the threshold of addiction. Exercise, including extreme sports such as bungee jumping, provided him with a ‘natural high’ and increased confidence.

Going bungee jumping and then wanting to do it a second, third, fourth, fifth, sixth time […]. I never did that in addiction. It's a natural high […]. It’s something that could get very addictive. […] The endorphins. […] Is better than any drugs. You'll build confidence […]. You'll feel great, you smile […]. I think it's so important for me in recovery […]. I'm not overdoing it. It's calculated. It's rewarding. My exercise isn't damaging to myself. Participant 4.

A participant in sustained recovery exercised intensively for 18 months in the third year of his recovery until suffering a back injury. While he was motivated by the improvement of his physical appearance and affirmation from females, exercise was also being used to avoid real-life issues. He conceptualized his actions as part of a pattern of immoderate involvement in behaviors.

I found myself very big, very big, very fat. Because I wanted to eat everybody’s plate finished (laughs) […]. And then I started exercising and then […] over-exercising and then I messed up my back. So yes, I always go overboard, with everything […]. I haven’t actually been to the gym, […] this whole year […]. I would probably still be gymming […]. To be out of my head man […]. So, I don’t have to think about my, my reality. Yes. Participant 14.

Another participant, who believed that he had temporarily substituted his SUD with exercise stated that his involvement with Mixed Martial Arts (MMA) escalated rapidly. He attributes his high engagement to finding a purpose for himself such that when he was assigned his placement for his medical training, he abandoned the exercise.

I got back into MMA. Very quickly I was like beating myself up for not going six times a week. I was close to within the first month accepting an amateur fight. […] I haven't trained in ages and […] I switched […] I found out about my placement at (hospital) and […] then had a purpose. […] I was just in recovery […] I was latching onto anything. […] I can […] get worth there. […] That was probably about three weeks. Participant 7.

In another account, a participant cycled and ran six days a week until, at the insistence of her sponsor, she reduced her exercise. Her exercise schedule, coupled with long work hours led her husband and sponsor to express concern about her well-being.

I'm training for the Argus (Cycle Tour) I've entered twice before and haven't finished. […] I've also joined a running program […]. It was six weeks I did it six times a week and that's too much. […] I was sick. Sick, sick, sick […] and my husband said to me: […] ‘You cycled and ran today. You haven't had a day off, you're working 10 h at work. You are doing too much.’ […] And […] my sponsor […] said ‘You look terrible, why are you so tired? What's going on?’ […] ‘Wednesdays off, Fridays off. That's it’. Participant 10.

Lastly, a participant that previously played basketball nationally felt that his gymming had become ‘obsessive’. Now, in the first three months of his recovery, he sought to recreate his earlier physical fitness level. He disregarded medical advice and despite injury resumed exercise earlier than recommended.

The month and a half after that, I was really obsessing about going to the gym. […] I wanted to be like before […], I played basketball on a national level. […] I still go to the gym sometimes but not every day anymore because […] it was really obsessive behavior. […] In that period, I also had an injury […] I had to rest for six weeks and after two weeks I was already in the gym. Participant 17.

### Pornography, sex and relationships

Pornography viewing, sexual activity, and relationships (*n* = 5) also came to the fore during recovery. One participant described the interplay of his SUD, sexual activity with sex workers, and overwork. Now that he had abstained from his primary substance, his sexual activity, which had formed part of his addiction set was starting to impact his marriage as well as his work. His sex addiction was more private, with his wife only aware of his pornography viewing. He was distressed about the double life he was leading as a Christian and identified that the sexual activity was placing him in harm's way – one example of which was fatigue at work, where he operated dangerous machinery (“[…] people … died on the job already, in my department […] I put my life at risk. […]”). Work was also being used as an explanation for his absence from home when he was engaging in extra-marital sex. His pornography consumption and sex dominated to the extent that he expressed: “My substitute addiction became my primary addiction”. He anticipates that his sexual activity will ultimately lead to relapse.

My sex addiction, it's private. […] My sponsor suggested that I go to SAA (Sex Addicts Anonymous) meetings […]. I went there, and […] thought […] I'm not as sick as this mense (people) […] but […] ek is (I am), […] I can't go back there, because I'm not ready to admit to my wife […]. She thinks it's only porn […], but then […] I am involved with other things. And, I know it’s a matter of time before it takes me back to my first addiction. And for a long time now I haven't gone to houses, […] but it will never stop. […] It stops for one week […]. You can’t pray and you feel overwhelmed […]. I know it's gonna fuck up my whole life […]. You are now probably the first person that I really—like, even the people in NA […] don't know, […] one of the reasons I, I'm not […] connected anymore […] (it) takes me to dodgy places sometimes, and I'm putting my life at risk. Participant 20.

An obsessive preoccupation with romantic relationships (ostensibly love addiction) may also be used to fill a perceived void or to derive self-worth.

A hole, there was like a part missing. I still have that feeling. And she kinda filled that part. She made me feel I meant something. […] I was obsessing about her every day […] I was checking if she was online […] because she didn’t answer I felt rejected, and then I got angry, so I see my cycle now […]. I’m still struggling, but it’s not bad. […] I realize it when I’m going in that behavior. […] I give my phone to […] my friend, or put it in my room […] or do something else. Participant 17.

Sex as a substitute may encompass pornography viewing or sex-based relationships, which may be used for distraction. Self-perceived pornography-related dysfunction may motivate abstinence.

Yes, I probably picked my partners because of the availability of sex […]. It was just like how can I distract myself and that was probably the best way to do it. […] Yes, in early recovery I got into a lot of relationships. […] I've been without a relationship for […] two and a half years. […] I'm trying to be comfortable with myself and to find out that I'm enough. […] I struggle with intimacy. Participant 5.

### Binge-watching series or pornography

A few participants (*n* = 3) noted that binge-watching series or pornography was a way to isolate themselves, avoid emotional pain, and escape reality.

If I'm angry and I want to isolate I can watch a whole series […], the entire weekend […], I'll call in take out, I won't even cook. I will just stay there in my room just watching […]. To avoid people. […] When I had really bad time or when my daughter emigrated, and I didn't want to deal with that pain. And I just started a whole lot of series because then I don't have to think. So, it is a way of escape. Participant 10.

### Excessive time spent working

 Periods of overwork were described by participants (*n* = 3) for reasons such as relapse prevention, escapism, and compensation for losses in ‘active addiction’.

I was working extensive hours […] 47 h overtime […] I still carry that title […] ‘Overtime King’. (laughs). […] Though I was performing at work, at home I just want to sleep […]. I am tired… nasty […]. My family started suspecting me of using because the behavior is the same […]. They made me permanent at work […]. It scaled down a bit, but […] didn’t change […]. From 7 ‘o clock the morning ‘till 11 ‘o clock at night. […] People […] say I’m trying to impress the boss, but […] my substitute addiction still makes my life unmanageable. Participant 20.

### Buying and shopping

The participants that reported in-store buying and shopping as a substitute (*n* = 2) describe it as a compulsive behavior that may have been present during ‘active addiction’, and endured during recovery, or, that it may have been initiated in recovery. In the first illustration, significant debts were incurred (20 000 ZAR/ $1102,37 US at the time of writing). To engage in shopping the participant waited until she was alone at home, arranged transport to the mall, and ensured that payment notifications would not reach her husband. It was only when she had exceeded her credit card limit that he became aware of her spending and advised her to keep within the budget. Furthermore, peers in NA challenged her as to how often she was discussing shopping (*“you are talking too much about shopping”*), which prompted her to reflect upon what was underlying her behavior.

I'd go to the shopping, you know, with money that I don't have […] and I could see it progressing […]. I knew it wasn't a positive effect in my life. […] I try to fill a void with something then I go shopping […] and I think to myself:’Why do you need that specific thing? What is going on inside here?’ […] ‘Is it anxiety? Is it fear? Is it, is it loss of something?’ […]. Over 20 grand—I maxed it out within a month […]. It was so easy. Participant 2.

The second participant illustrates that the excess money that was ordinarily allocated to purchasing substances was immediately used for shopping. Perceived benefits of shopping included: a rewarding feeling and improved view of the self; the ability to make others happy; ridding oneself of surplus money and eliminating an uncomfortable feeling state. However, the behavior was described as being unmanageable.

It's a coping mechanism that allows you to feel better about yourself. […] But the urge just to spend money it’s crazy. It's unmanageable. Completely. […] It wasn't always me that had to benefit from all the shopping. […] I wanted to make everybody else happy. […] Now that I'm not inclined to go buy drugs, I will buy sweets […]. The money will burn holes in my pocket. […] That feeling that I get rid of when I spend money or when I get rid of the money. […] It's much more rewarding. Participant 9.

Having elucidated the potential manifestations of substitute behaviors and addictions, participants also shared their insights on the nuanced issue of harm.

## Substitute behaviors and harm (reduction)

Participants believed that while the nature of the substitute behavior was a key consideration in establishing its potential to harm, behaviors that are ostensibly healthy or supportive of recovery could also lead to relapse. It was considered essential to recognize patterns in this behavior and to be able to determine whether the substitute made life unmanageable. It was believed that substitutes may threaten recovery by leading to relapse and by eliciting the same feelings; leading to losses and requiring dishonesty to maintain as did the abstained addictive behavior. A lack of knowledge of the dynamics of a substitute behavior could also harm.

These quotes express that relapse may gradually occur if a substitute becomes compulsive and works against the gains of recovery to make life unmanageable (again). The threshold for determining whether a behavior is harmful was said to be its effects on the self and relationships.

If it becomes compulsive behavior […] ruins things that you applied in recovery. […] Becomes aggressive and you forget about other things within yourself […]. That’s the thing for relapse. Participant 2.

You can’t stop yourself […] you are obsessing […] which makes your life unmanageable. […] resentful, angry […] ashamed, anything that’s gonna make you feel like less. That is, you are still acting out on your addiction. Participant 18.

These quotes show that the void of the terminated addictive behavior may be filled with a range of behaviors, with not all being harmful or addictive. However, ostensibly healthy or unhealthy behaviors engaged by persons with a history of addiction were potentially harmful as they could be justified and continue.

I mean if you had the choice between being a heroin ‘addict’ or a Comrades (marathon) runner, I’d rather go for a Comrades (marathon) runner […]. You are so used to having your soul, your identity’s consumed by being an ‘addict’. You take that out, what's left? Sometimes the easiest way out is to give that person something. ‘Okay you can't have heroin but here, have a cup of coffee’. So, I don't think it's always (harmful), but I mean the majority of the time, yes. […] There are some guys pursuing all kinds of weird things to keep them clean. Participant 4.

One participant reported that whether a behavior is regarded as healthy or not, it was always harmful in excess and that it was always negative to substitute one substance with another.

I think excess is always harmful. I mean even if it's something healthy like exercise. […] I can't think of a single example where substituting with a substance would be a good idea. […] The behaviors […] you can convince yourself a lot easier that it's healthy. Participant 7.

According to other participants, a substitute engaged in excess was harmful as it prohibited persons in recovery from being present. Furthermore, any excessive behavior was considered harmful or even potentially fatal.

Anything excessive could harm you. So, even if it's exercise. You’re literally not dealing with the fact that you can't handle your emotions and your well-being on a level that's balanced. […] I do believe that in excess anything could kill you. Anything. Even denial. Participant 9.

Participants also reported that the behavior underlying the varied manifestations of addiction is more important than the addictive behavior itself. If persons in recovery could recognize patterns in behavior, and when they become ‘obsessed’, the disease itself could be addressed, and substitutes don’t need to arise.

Addiction is addiction […] it’s the disease of more. […] Sometimes it changes, uhm but it’s the behavior that needs to be focused on. […] When I become obsessed, does my thinking follow a pattern? […] Make a start recognizing this pattern. […] If you can deal with the disease itself, there shouldn’t be several addictions. […] A lot of the time […] people came to replace their active addictions with healthier things […], with exercise […] family life […] raising children […] it doesn’t necessarily have to be toxic. Participant 13.

Participants expressed that substitutes could be detrimental to recovery via various processes. Substitutes may elicit the same feelings as did the abstained addictive behavior; may lead back to the primary substance or, lead to comparable losses.

Gambling is going to strip you of everything as the drugs did. […] still going to eat your money too because you're not always going to win. So, it can be harmful because you can lose your home […] your family […] everything. Participant 16.

It takes me back to that feeling of how I felt when I was in active. […] (I) stop being honest with myself. […] Definitely, it doesn't work. […] I don't think it really matters […] what the addiction is […]. It's harmful to me because I’ll get to a point where I'll say to myself it's okay? Participant 23.

Finally, one participant spoke about the sub-cultures and paraphernalia linked to certain drugs of choice and the potential dangers of lacking ‘expertise’ in the aspects and dynamics of the substitute behavior, leading to harm.

## Support needs to manage and resolve substitute behaviors

NA members offered a range of recommendations for managing substitute behaviors. Specifically, the importance of continued engagement with recovery support and the need to be educated about changes in patterns of behaviors that could progress to become substitute addictions were noted. Furthermore, participants advised service providers to focus on and process the underlying emotional states that may underpin addictive behaviors and add to or develop a repertoire of adaptive coping skills. Service providers were urged to establish whether co-occurring addictive behaviors or disorders are present as these pose a risk for relapse and substitution.

Ongoing engagement in recovery support programs was considered necessary as the potential for substitute behaviors to arise would always be there.

If an ‘addict’ doesn't stay in the program and find daily relief […] they will cross-addict […] manipulate, lie […] cover up […] another addiction. […] Rationalize to themselves and to everyone else […] that they are not addicted. It might start off slowly, quickly […] progress. Participant 11.

Speaking to behavioral addictions specifically, it was expressed that people who use substance use treatment services should be educated in a highly practical way about what would constitute healthy as opposed to excessive engagement in a behavior. In the case of behaviors, some may be carried over from ‘active addiction’ into recovery, and these should be addressed in the long term:

When it comes to process addictions […] what's carried over, what has changed from the past? […] Education […] needs to […] become bit more practical in recovery. What is a good amount of time to exercise? […] What is a healthy meal plan? […] What is a healthy exercise routine? […] What entails a healthy sexual life? […] What’s normal? […] We have no comprehension of what normal is. Participant 4.

As it was believed that the manifestation of the addiction could change, people who use services should be taught to identify patterns in behavior. Furthermore, practitioners should seek to explore and educate people who use services as to why they sought out substance use initially (its functions), and work towards capacitating them to confront underlying emotions. One participant assigned a higher value to insights shared by persons in recovery, as they were said to better understand lived experiences than a trained professional.

You must fight the underlying emotional state […]. It doesn't matter what you are addicted to. […] It's about what you’re trying to hide. It's what you’re trying not to see […]. Teaching […] how to emotionally capacitate themselves rather than trying to push down an addiction […]. Why they wanted something as a substance to use in the first place. Participant 9.

Addiction for me is coping with pain […]. ‘Addicts’ […] need to learn the, the normal way of coping. […] The thing about NA […] if they tell me something out of their experience, maybe it works for me. […] Sometimes if someone like my psychologist […] would say: […] ‘try that’ […] What do you know? […] You don’t know how my mind works. Participant 17.

To promote stable recovery and prevent relapse it was deemed vital by participants to explore and assess potential concurrent addictive behaviors and disorders. Moreover, it is necessary to establish whether a mental health condition coexists with an addictive disorder, and may precipitate relapse when left unmanaged. The importance of aftercare was also noted. Finally, participants encouraged providers to explore and prioritize secondary behaviors alongside the SUD, as these may intensify and become unmanageable in time.

If someone says they’re engaging in a behavior but the behavior isn’t so serious […]. Encourage the person to really work on that thing […]. Because that's the next thing that he’s gonna focus on […]. I have this porn addiction […] I spoke to my counselor about it, but for me it was, you’re here for this (drugs) – ‘let’s now just focus on this’. But, actually, we needed to focus on that other thing also […] and try to get balance on it […] that thing is going to grow. Participant 20.

## Discussion

NA attendees in South Africa described that substitute behaviors (including cigarettes, food, sex, and exercise) developed across various recovery stages. Substitute behaviors could be temporary or long-term replacements for SUDs utilized for distraction, isolation, mood modification, harm reduction, or relapse prevention. Yet, while substitutes could decrease harm, it was believed that even ostensibly healthy behaviors could threaten recovery.

Given that abstinence-based recovery has historically excluded nicotine and caffeine [3], it is perhaps unsurprising that these emerged as common substance-based substitute behaviors among participants. Reich and colleagues [40], in their study of 289 AA recovery support group members in the USA, found that levels of coffee and nicotine consumption exceeded that of the general population. Moreover, the quantities consumed were also larger amounts per capita. While negative affect reduction was reported by study participants as a motive for smoking, use was also attributed to the availability of money and reducing boredom. The role of cigarettes in recovery remains hotly debated with evidence of its link to relapse [41] and smoking cessation and improved substance use outcomes [42]. The continuation and escalation of cigarette consumption [3] among NA participants highlight that smoking cessation should be a task in early recovery [43].

Findings from the in-depth interviews suggest that although common in early recovery, substitutes arose at all stages of recovery. In one of the few such studies in the South African and broader African context, Stokes and colleagues [31] conducted individual, face-to-face interviews to shed light on how participants’ sustained recovery was achieved. Affiliation with a 12-Step program and acceptance of the chronic ‘disease’ concept was found to support stable recovery [31]. Insofar as this concerns substitute behaviors and the narratives of participants in the present study, this may relate to how sponsors or fellow recovery support group members would raise concerns about (potential) substitute behaviors, which would prompt reflection and, in some cases, action. Participants also emphasized the disease model and used it as the basis for belief in different manifestations of the disease and as a motive for accessing recovery support. However, this perspective aligns more closely with The Syndrome Model of Addiction [44], which asserts that those with the syndrome are susceptible to substance- or behavior-based addictive behaviors.

Substitute behaviors have been discussed in terms of their instrumentality in fulfilling heterogeneous motives. The motives for substitution expressed by participants align with and extend earlier research. While the use of substitutes for time-spending, harm reduction, relapse prevention, and coping, broadly construed, is commonly known [16, 22, 45], participants also identified self-soothing, distraction, escapism, and avoidance as motives. It is interesting to note that different substitutes fulfilled these same functions and that different motives could underpin the same behaviors. These findings may also highlight specific areas for building and developing alternative behaviors that represent adaptive coping. The extent to which individual expectations are met and may reinforce the behavior may lead to continued engagement or use of the substitute. However, participants’ narratives also exhibit that while the possibility remains that temporary substitutes may become long-term replacements, substitute behaviors can be time-limited [46].

In this study, participants asserted that behaviors, not all of which could be abstained from (e.g. eating), needed vigilance and ongoing management. Accordingly, unmanageability or continued engagement in the face of harm to oneself or relationships was said to indicate when a substitute behavior was becoming a substitute addiction. Griffiths [47] argues that while acknowledging that “loss of control” is defining of addiction for most, behavioral addictions in particular (e.g., work addiction) may present without loss of control. Yet, “control (and loss of it) may be something that changes its nature over time” ([47], p. 2–3). Furthermore, it is noteworthy that some participants used highly stigmatizing, pejorative language to describe people in recovery, and themselves (e.g. Participant 11). It is plausible that self-stigma can discourage disclosures about the presence of and potential impact of substitute behaviors on health and recovery.

Multiple addictive behaviors were two potential threats to recovery that were discussed by participants. International [48] and South African studies [49] of persons receiving treatment for SUDs have demonstrated the co-occurrence of behavioral addictions. In the USA, a sample of 51 people receiving substance use treatment reported a variety of co-existing behavioral addictions including sex/pornography; eating; shopping/spending; work; computer/internet; exercise; gambling, and self-harm. Such multiple addictive behaviors are known to make the presentation and intervention more complex [49]. The manifestations of interacting addictive behaviors vary and cause more harm than solo addictions [34]. In the present study, one pattern of interactions entailed a secondary substance or behavior escalating once the primary problem was abstained from (e.g., cigarettes), or one behavior was used to mask another (e.g., purporting to work long hours to engage in sex).

Participants described that feedback from sponsors and group members during meetings were features of NA that aided the identification and management of substitute behaviors. Sponsorship, having regular exchanges with a peer with more time in recovery, is a central element of NA. Sponsors may listen to concerns and offer support, provide direct, honest feedback and, at their discretion, share their experiences [50]. Keeping contact and having a strong sponsor-sponsee alliance has been shown to predict abstinence and recovery support group participation [8]. NA and other 12-Step meetings provide a forum to share recovery experiences and rely on the “therapeutic value” of one person with lived experience of ‘active addiction’ helping another [51], an aspect particularly valued by some study participants. As experienced members and newcomers alike share in meetings, emerging or established substitute behaviors may be seen in a new light.

Participants highlighted the importance of subjective experiences of substitution, and how what may be regarded as a problem, may differ. All substitutes reported in this manuscript are those regarded by participants and the primary researcher as substitute behaviors. Some participants recognized behaviors that predated their recovery and endured, to be substitute addictions. However, others did not consider certain of their behaviors to be substitution, as it was maintained at the same level as in ‘active addiction’. Notwithstanding individual experiences, we contend that behaviors that are ‘held’ at the same strength as in ‘active addiction’ still potentially constitute substitute behaviors as they could still fulfill the function(s) of a terminated behavior. The comparative harm of substitute behaviors may also differ. Taken together, these findings are important and relevant to recovery as substitutes may precipitate relapse, impact functioning, or reduce harm [52].

### Limitations

While this study addresses the need for research on substitution within recovery support groups, its limitations center on being conducted only in the Western Cape province. Increasing the diversity of the sample may facilitate transferability to other settings. It is also noteworthy that all but four participants had received formal treatment for their SUD(s), some of whom had multiple treatment episodes. Future research should be conducted among NA members in other settings, and further qualitative and quantitative research should examine substitute behaviors in stable and sustained recovery and with members of other 12-Step programs. The data analyzed here also suggest that future longitudinal studies would be beneficial to explore how substitute behaviors are managed over time. Finally, the findings of this study suggest that levels of recovery capital, the personal, familial/social, and community assets that support recovery [5] merit further consideration as to its role in substitution beyond our formative work with people receiving substance use treatment [45].

## Conclusions

Based on interviews with NA attendees various clinical implications emanate from the study. First, it is vital for persons in recovery to establish whether they have multiple addictive behaviors that need to be addressed within a relapse prevention plan. Second, and given that recovery support group attendees may also engage in formal treatment, service providers should offer practical guidance on self-monitoring to aid prevention, identification, and management of substitute behaviors as well as indicators of harm. Particularly in the case of behaviors, not all of which can be abstained (e.g., food), those that continue from ‘active addiction’ into recovery, should be monitored and managed in the long term. Third, those in recovery should be informed of the different pathways along which substitutes can arise.

## Supplementary Information

**Additional file 1.**

## Data Availability

The datasets generated and analyzed during the current study are not publicly available due to individual privacy and confidentiality but are available from the corresponding author upon reasonable request.

## Abbreviations

NA: Narcotics Anonymous
SUDs: Substance use disorders
AA: Alcoholics Anonymous
LMICs: Low- and middle-income countries
SAA: Sex Addicts Anonymous
MMA: Mixed Martial Arts

## Footnotes

1 The racial categories ‘Black African’, ‘Coloured’ and ‘Asian/Indian’ originate from apartheid South Africa where it designated those denied the same benefits as ‘Whites’, and reinforced segregation. We use these terms here only for descriptive purposes, given the importance of continued redress efforts and as they are referred to in the South African context.
