# Supplementary material for: Narcotics Anonymous attendees’ perceptions and experiences of substitute behaviors in the Western Cape, South Africa
Source: Subst Abuse Treat Prev Policy. 2023 Jul 5;18:40. doi: 10.1186/s13011-023-00552-z (PMC10324242; doi:10.1186/s13011-023-00552-z)
Supplement: Supplementary file 1 — Additional file 1. [file 13011_2023_552_MOESM1_ESM.docx]

**In-depth Interview Schedule**

Thank you for your time today. In this interview, I’d like to talk to you about your recovery experience and experiences pre-recovery.

1. Could you tell me a bit about yourself?
   1. How old are you?
   2. Are you employed? If retired, what was your profession previously?
   3. Are you married or in a relationship?
   4. Are you a parent?
   5. What would you like for me to know about you before we start this interview?
   6. Which substances have you used?
2. What does recovery mean to you?

***Brief history of substance use and treatment***

For some individuals, recovery starts with a formal treatment program.

1. Have you ever been treated for substance use?
2. For which substance/s were you treated?
3. For how long were you using ____________?
4. For how long were you in treatment? (Repeat for each treatment episode)

c. Did you complete the treatment program?

- (If yes): What enabled you to complete treatment?
- (If no): What do you think prevented you from completing the treatment program?

1. How do you think you’ve managed to stay clean?
2. Is there any behavior that helps you stay off _____?
3. Do you have any relapse prevention plan in place for yourself? Please tell me more about that.
4. Do you have an aftercare plan in place for yourself? Please tell me more about that.

If the support group is mentioned/Interviewee states: You belong to a recovery support group:

1. What do you gain from belonging to the recovery support group?
2. What role does the support group play in your life?
3. What does your involvement in the support group involve?
4. Are there any positive aspects of belonging to a support group?
5. Are there any negative aspects of belonging to a support group?

***Recovery and substitute addiction***

1. How would you describe your life in recovery from _____________? (e.g. self, relationships)
2. Since you’ve been in recovery, have you made any lifestyle changes?
   1. What have those changes been?
3. Since you first pursued recovery, have you noticed yourself doing any behaviors or activities over and over again/ or more of/ that you didn’t do before?
4. Are these behaviors that you’re happy or unhappy with?

Probe around use of *substances:* Alcohol; Nicotine/cigarettes; CAT; Cocaine/crack; Cannabis; Cannabis/Mandrax; Ecstasy; Heroin; Inhalants; Methamphetamine; Nyaope/Whoonga; Over-the-counter and prescription medicines?

Probe around the following *behaviors:* Exercise; Shopping; Sex; Eating; Work; Love/ relationships; Religious activities; Use of internet and video games; Social networking (e.g. Facebook) and/or Gambling? Please tell me more about that.

**If the interviewee responds yes to questions 13 or 14, ask:**

1. Please could you tell me more about how you started using ________/ or engaging in __________/used more of/ engaged in _________ more than before?
2. What made you gravitate toward this behavior?
   1. How **accessible** was the substance or behavior to you?
   2. What made the behavior **appealing** to you?
   3. Was there any **specific language** you needed to be familiar with to access the substance/behavior?
   4. Who did you need to **communicate** with to access ____ or perform the behavior_______?
   5. Did you experience any **feelings of belonging** with activities and groups related to the behavior?
   6. What were your **expectations** of __________? How well did the behavior **meet your expectations**?
3. Have you experienced any **advantages or disadvantages** due to your increase in ____________?
4. Do you think that you’ve substituted your addiction to ______________?
5. What are your **thoughts on substitution**?

**If interviewee hasn’t had personal experience of substitute addiction, ask:**

1. Does stopping one addiction place someone at risk of developing a new addiction? Please elaborate.
2. Is it always harmful to replace one substance with another/ engage in a new behaviour excessively? Please elaborate.
